# 帕博利珠单抗对晚期非小细胞肺癌患者T淋巴细胞亚群的影响及疗效观测

**DOI:** 10.3779/j.issn.1009-3419.2021.103.03

**Published:** 2021-03-20

**Authors:** 芸 王, 郁阳 王, 曼 姜, 月妹 赵, 晓春 张

**Affiliations:** 1 266003 青岛，青岛大学附属医院肿瘤科 Department of Medical Oncology, The Affiliated Hospital of Qingdao University, Qingdao 266003, China; 2 261000 潍坊，山东省潍坊市人民医院 Weifang People's Hospital of Shandong Province, Weifang 261000, China

**Keywords:** 肺肿瘤, 帕博利珠单抗, 免疫治疗, T淋巴细胞亚群, Lung neoplasms, Pembrolizumab, Immunotherapy, T lymphocyte

## Abstract

**背景与目的:**

本研究旨在探究非小细胞肺癌患者应用帕博利珠单抗治疗前后外周血淋巴细胞亚群的变化及临床意义。

**方法:**

选取2015年1月-2020年12月至青岛大学附属医院及潍坊市人民医院行帕博利珠单抗治疗的32例非小细胞肺癌的患者为观察组，另外选取同期30例健康查体者为对照组。治疗前、治疗后第1、2、4周期，采用流体细胞术检测患者外周血淋巴细胞亚群水平的变化。

**结果:**

非小细胞肺癌患者治疗前的CD3^+^、CD4^+^、CD4^+^/CD8^+^指标较对照组明显下降（*P* < 0.05），CD8^+^水平明显升高（*P* < 0.05）；应用帕博利珠单抗治疗1个周期后，淋巴细胞亚群较免疫治疗前的变化未见明显统计学差异；治疗2周期后CD3^+^、CD4^+^、CD4^+^/CD8^+^数值较治疗前升高（*P* > 0.05），CD8^+^指标较治疗前下降（*P* < 0.05）；治疗4周期后CD3^+^、CD4^+^、CD4^+^/CD8^+^数值较治疗前得到显著改善（*P* < 0.05），CD8^+^指标较治疗前明显下降（*P* < 0.05）；疗效达到疾病稳定（stable disease, SD）/部分缓解（partial response, PR）患者在治疗过程中，第4周期的CD3^+^、CD4^+^、CD4^+^/CD8^+^数值较治疗前升高（*P* < 0.05），CD8^+^指标较治疗前降低（*P* < 0.05）；疗效达到疾病进展（progressive disease, PD）患者在治疗过程中，淋巴细胞亚群变化较治疗前均没有明显统计学差异（*P* > 0.05）。同时本文通过分析显示，程序性死亡受体配体1（programmed cell death ligand 1, PD-L1）表达与否及病理学类型对免疫治疗效果的影响不明显。通过多因素分析显示，同时观察CD3^+^、CD4^+^、CD8^+^的变化对预测免疫治疗效果更有意义。

**结论:**

帕博利珠单抗可以调节非小细胞肺癌患者T淋巴细胞亚群的变化，改善患者机体的免疫状态，且未见明显不良反应，同时治疗过程中监测淋巴细胞亚群变化可预测免疫治疗效果。

## Abstract (en)

**Background and objective:**

The aim of this study is to investigate the changes of peripheral blood lymphocyte subsets before and after treatment with pembrolizumab for non-small cell lung cancer and its clinical significance.

**Methods:**

A total of 32 patients with non-small cell lung cancer who received pembrolizumab treatment in The Affiliated Hospital of Qingdao University and Weifang People's Hospital of Shandong Province from January 2015 to December 2020 were selected as the observation group, and 30 healthy people during the same period were selected as the control group. Before treatment and in cycles 1, 2 and 4 after treatment, fluid cytometry was used to detect changes in the levels of lymphocyte subsets in the peripheral blood of patients.

**Results:**

The CD3^+^, CD4^+^, CD4^+^/CD8^+^ indexes of patients with non-small cell lung cancer before the treatment were significantly lower than those in the control group (*P* < 0.05), and the CD8^+^ level was significantly increased (*P* < 0.05); After 1 cycle of pembrolizumab treatment, there was no significant difference in the changes of lymphocyte subsets compared with before immunotherapy; After 2 cycles of the treatment, the CD3^+^, CD4^+^, CD4^+^/CD8^+^ values were higher than before the treatment (*P* > 0.05), and the CD8^+^ index was slightly lower than before the treatment (*P* < 0.05); After the fourth cycle of treatment, the CD3^+^, CD4^+^, CD4^+^/CD8^+^ values were significantly improved compared to before the treatment (*P* < 0.05), and the CD8^+^ index was significantly lower than before the treatment (*P* < 0.05); In the treatment process of patients with stable disease (SD)/partial response (PR), the CD3^+^, CD4^+^, CD4^+^/CD8^+^ values of the fourth cycles were higher than before the treatment (*P* < 0.05), and the CD8^+^ index was lower than before the treatment (*P* < 0.05); During the treatment of progressive disease (PD) patients, the changes of lymphocyte subsets in the fourth cycles were not significantly different from those before the treatment (*P* > 0.05). At the same time, this article shows through analysis that the expression of programmed cell death ligand 1 (PD-L1) and pathological types have no obvious influence on the effect of immunotherapy. Multi-factor analysis shows that it is more meaningful to observe the changes of CD3^+^, CD4^+^ and CD8^+^ at the same time to predict the effect of immunotherapy.

**Conclusion:**

Pembrolizumab can regulate the changes of T lymphocyte subsets in patients with non-small cell lung cancer, improve the immune status of the patients, and there is no obvious adverse reaction. At the same time, monitoring the changes of lymphocyte subsets during treatment can predict the effect of immunotherapy.

肺癌是世界上与癌症相关死亡的最常见原因，而非小细胞肺癌占原发性肺恶性肿瘤的80%左右，只有肿瘤完全切除的患者才有很大的机会增加生存率，但是多达30%的Ⅰ期疾病患者术后会复发^[1]^。肺癌的发生、发展与宿主的细胞免疫、适应性免疫和固有免疫密切相关，其中以T淋巴细胞为主导的细胞免疫在抗肿瘤的免疫反应中发挥着重要作用^[2]^。肿瘤微环境的免疫抑制性降低了淋巴细胞的浸润，导致机体免疫功能紊乱，有利于肿瘤细胞的免疫逃逸，因此淋巴细胞亚群的变化可用于监测和判断肿瘤患者的病情与预后^[2]^。

免疫治疗已成为系统性癌症治疗的额外选择，而且往往是更好的选择。程序性死亡受体1（programmed death 1, PD-1）在活化的T细胞、B细胞和骨髓细胞中表达^[3]^。在正常情况下，PD-1与其配体（programmed cell death ligand 1, PD-L1）的结合可拮抗主要组织相容性复合体（major histocompatibility complex, MHC）-CD3介导的T细胞活化途径，防止由于T细胞过度活化而引起的组织损伤和炎症，因此肿瘤细胞通过高表达PD-L1来逃避T细胞的杀伤作用^[3, 4]^。PD-1抑制剂与免疫检查点结合，可以重新激活T细胞的免疫反应以及抑制免疫抑制性调节性T细胞，进一步激活宿主免疫系统以识别、攻击和根除肿瘤细胞，已被认为是临床治疗中一种有前途且有效的策略^[3, 5]^。该疗法已显示出在治疗肺癌方面的持续临床反应，但疗效各不相同，部分取决于肿瘤浸润淋巴细胞的数量和性质，目前应用于非小细胞肺癌治疗的帕博利珠单抗受到广泛关注^[5]^。因此，本文通过分析帕博利珠单抗治疗的非小细胞肺癌患者的外周血中淋巴细胞亚群变化以及治疗过程中不同疗效患者外周血淋巴细胞亚群的变化，讨论应用帕博利珠单抗治疗对非小细胞肺癌患者外周血淋巴细胞亚群的影响。

## 1 资料与方法

### 1.1 一般资料

选取2015年1月-2020年12月至青岛大学附属医院及潍坊市人民医院就诊的32例Ⅱ线单药应用PD-1免疫抑制剂帕博利珠单抗的非小细胞肺癌（Ⅳ期）病例为观察组。本次研究的所有病例都经过病理学明确诊断，其中肺鳞癌25例，肺腺癌7例，在本次研究的非小细胞肺癌患者中，PD-1表达均为阳性。与此同时，选取了30例同期健康查体人员作为对照组，两组的基线数据见表 1。

**1:** 患者临床特征 Clinical characteristics of patients

Features		Healthy people	NSCLC	*P*
Gender	Male	18	19	0.326
	Female	12	13	
Age(yr)	>60	7	10	0.083
	< 60	23	22	
Smoking status	Ever	11	25	0.043
	Never	19	7	
NSCLC: non-small cell lung cancer.				

### 1.2 排除标准

免疫系统、血液系统疾病的患者；近期存在明显感染的患者；无可测量病灶的癌症患者。

### 1.3 研究方法

#### 1.3.1 检测方法

所有患者采集静脉血2 mL，DETA-K2抗凝，进行外周血淋巴细胞亚群的检测。采集操作均由经验丰富的检验科技师完成。

#### 1.3.2 近期疗效观察

根据实体瘤疗效评价标准（Response Evaluation Criteria in Solid Tumors, RECIST）1.1指南，疗效标准为：完全缓解（complete response, CR）：所有目标病灶完全消失；部分缓解（partial response, PR）：所有可测量的目标病灶的直径总和低于基线≥30%；疾病稳定（stable disease, SD）：基线病灶直径总和有缩小趋势，但是未达到PR的标准，或有增加但是未达到疾病进展（progressive disease, PD）的评价标准；PD：目标病灶的直径总和增大20%超过基线或者出现新的病灶。本文中所有患者的疗效评价均通过影像资料参照疗效评价标准进行评价。

### 1.4 统计学方法

使用SPSS 22.0及GraphPad Prism 8.4.3对实验数据进行统计分析。计数资料用*χ*^2^检验，计量资料用均数±标准差（Mean±SD）表示，比较用*t*检验，同时用*Logistic*回归行多因素分析，*P* < 0.05为差异具有统计学意义。

## 2 结果

### 2.1 观察组帕博利珠单抗治疗前淋巴细胞亚群水平与对照组淋巴细胞亚群水平比较

观察组32例非小细胞肺癌患者治疗前的CD3^+^、CD4^+^、CD4^+^/CD8^+^指标较对照组明显下降（*P* < 0.05），CD8^+^水平明显增高（*P* < 0.05）。同时，比较了吸烟与非吸烟者之间淋巴细胞亚群的变化差异，发现无统计学意义（*P* > 0.05）。可见，非小细胞肺癌患者机体处于免疫抑制状态，免疫功能较健康对照组低。

### 2.2 观察组患者治疗前后的淋巴细胞亚群变化

观察组32例患者免疫治疗前与治疗1个周期后淋巴细胞亚群水平比较，结果显示治疗1个周期后CD3^+^、CD4^+^、CD4^+^/CD8^+^数值虽然较治疗前升高，CD8^+^指标较治疗前稍有下降，但差异无统计学意义（*P* > 0.05）；治疗2周期后CD3^+^、CD4^+^、CD4^+^/CD8^+^数值较治疗前升高（*P* > 0.05），差异无统计学意义，CD8^+^指标较治疗前下降（*P* < 0.05），差异有统计学意义；治疗4个周期后CD3^+^、CD4^+^、CD4^+^/CD8^+^数值较治疗前得到显著改善（*P* < 0.05），CD8^+^指标较治疗前明显下降（*P* < 0.05）。患者行4个周期的治疗后外周血淋巴细胞亚群得到明显改善，比较各周期淋巴细胞亚群的变化差异发现，第2周期治疗后淋巴细胞亚群的变化与第1周期治疗后相比较，差异无统计学意义（*P* > 0.05），第4周期治疗后淋巴细胞亚群的变化与第1周期治疗后相比较，差异有统计学意义（*P* < 0.05）（表 2），而第4周期治疗后淋巴细胞亚群的变化与第2周期治疗后相比较差异没有明显的统计学意义（*P* > 0.05）。本文以CD3^+^、CD8^+^变化为代表绘制了变化图，见图 1。

**2:** 第1、4周期治疗后淋巴细胞亚群的变化 Changes of lymphocyte subsets after the first and fourth cycles of immunotherapy

Group	Treatment period	CD4^+^(%)	CD8^+^(%)	CD4^+^/CD8^+^	CD3^+^(%)
Observation group	Before treatment	31.42±6.87	36.13±9.08	0.65±0.29	53.31±12.17
	T1	29.92±10.66	32.08±8.38	0.79±0.38	59.15±17.93
	T4	38.97±8.45	26.98±8.16	1.31±0.64	68.70±12.72
T here briefly represents the period.					

**1:**
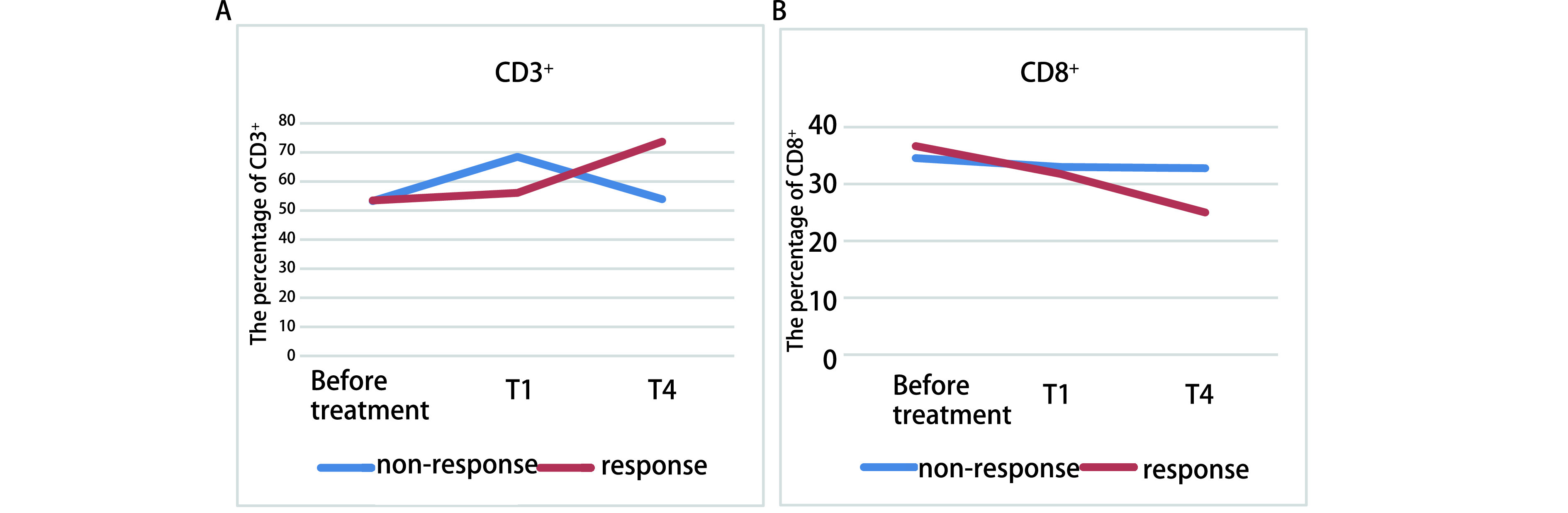
免疫治疗过程中CD3^+^（A）、CD8^+^（B）T淋巴细胞的变化趋势 The change trend of patients' CD3^+^ (A) and CD8^+^ (B) T lymphocyte subsets during immunotherapy, T briefly represents the period; response means SD/PR, non-response means PD.

### 2.3 观察组患者帕博利珠单抗疗效与T淋巴细胞亚群变化的情况

观察组32例非小细胞肺癌患者中，进行帕博利珠单抗治疗后第4周期后达到SD、PR的患者均为24例，占总观察人数的75%，达到PD的患者人数为8例，占总观察人数的25%。结果显示：第4周期免疫治疗后，疗效达到SD/PR患者的CD3^+^、CD4^+^、CD4^+^/CD8^+^数值较治疗前升高（*P* < 0.05），CD8^+^指标较治疗前下降（*P* < 0.05）；疗效达到PD患者的淋巴细胞亚群变化，较治疗前没有明显的统计学差异（*P* > 0.05），见表 3。PD-L1的表达阳性的患者与PD-L1表达阴性患者的免疫治疗治疗效果未见明显统计学差异（*P* > 0.05），肺鳞癌与肺腺癌患者在应用免疫治疗后的效果也未见统计学差异（*P* > 0.05），见表 4。

**3:** 第4周期治疗后不同疗效患者的淋巴细胞亚群比较 Changes in the relationship between lymphocyte subsets after the four cycle of immunotherapy

Curative effect		CD3^+^(%)	CD4^+^(%)	CD8^+^(%)	CD4^+^/CD8^+^
PR/SD		73.64±7.94	41.72±6.01	25.06±1.28	1.17±0.49
	*t*	6.82	7.25	5.1	6.71
	*P*	0.00	0.00	0.00	0.00
PD		53.89±13.22	30.71±9.67	22.43±6.04	1.99±0.60
	*t*	0.12	0.15	0.32	0.82
	*P*	0.91	0.88	0.76	0.44
PR: partial response; SD: stable disease; PD: progressive disease.					

**4:** 不同疗效患者的临床特征 Clinical characteristics of patients

Features		Non-response	Response	*P*
PD-L1 expression	>50%	5	14	0.165
	< 50%	3	10	
Pathology type	Lung adenocarcinoma	2	5	0.08
	Lung squamous cell carcinoma	6	19	
PD-L1: programmed cell death ligand 1.				

本文通过应用*Logistic*回归对多因素分析并绘制受试者工作特征曲线（receiver operating characteristic curve，ROC曲线）（图 2），结果发现CD3^+^、CD4^+^、CD8^+^淋巴细胞对免疫治疗效果具有一定的预测作用。

**2:**
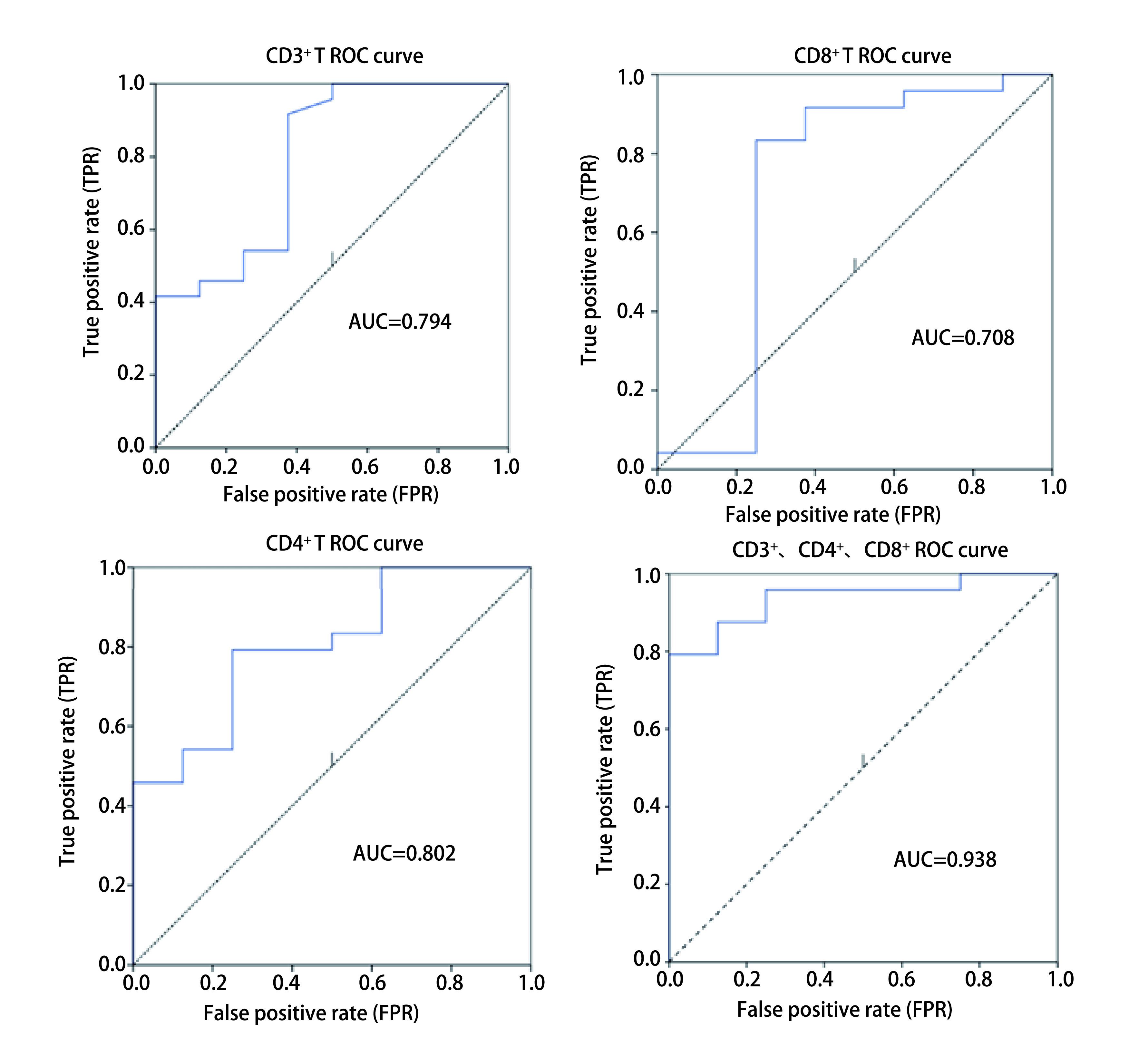
第4周期治疗后CD3^+^、CD4^+^、CD8^+^ T淋巴细胞的ROC曲线 The ROC curve of CD3^+^/CD4^+^/CD8^+^ T lymphocyte after the fourth cycle of immunotherapy. ROC: receiver operating characteristic curve.

## 3 讨论

肿瘤的防治一直是医学研究的重要方向。近年来，通过免疫治疗调动机体免疫系统，达到清除并杀灭肿瘤细胞的治疗方式取得了突破性进展。人体的免疫系统主要包括天然免疫和由体液免疫、细胞免疫组成的获得性免疫，其中细胞免疫被认为是机体抗肿瘤免疫的主要方式^[6]^。淋巴细胞大量浸润是细胞免疫发挥抗肿瘤反应的核心，同时也是免疫治疗发挥作用的细胞学基础^[6]^。因此，淋巴细胞亚群的监测对肿瘤的预防、诊断和药物疗效的判断具有重要的指导意义。淋巴细胞亚群中，根据其表面的标志物及生物学特性可以分为：T淋巴细胞、B淋巴细胞和自然杀伤（natural killer, NK）细胞，其中T淋巴细胞亚群是是淋巴细胞亚群的主要免疫应答形式^[7]^。T淋巴细胞的特有分子标志为CD3，存在于所有T淋巴细胞的表面，将其进一步细分可分为CD4^+^ T和CD8^+^ T细胞，其中CD4^+^辅助/诱导T细胞在抗肿瘤的免疫过程中处于核心地位，其分泌的细胞因子不仅对细胞免疫有正向增强作用，而且能促进B细胞的增殖，有利于抗体的产生，辅助体液免疫发挥抗肿瘤作用；CD8^+^ T细胞有两种表型一种是CD8^+^/CD28+，具有细胞毒作用，另一种是CD8^+^/CD28+，具有抑制作用^[7, 8]^。研究^[7]^表明，CD8^+^ T细胞不仅对抗原呈递细胞有一定的细胞毒作用，而且其产生的抑制性的细胞因子，能负反馈抑制CD4^+^ T淋巴细胞的表达，对体液免疫和细胞免疫有抑制作用，因此CD8^+^ T细胞的增多为肿瘤细胞的增殖和转移提供了条件。在正常情况下，CD4^+^/CD8^+^的比值处于相对平衡状态以维持机体的正常免疫反应，比值下降时说明机体的细胞免疫功能下降，对肿瘤细胞的杀伤作用减弱，使患者机体处于免疫抑制状态。

本研究发现，与健康查体的对照组相比较，所观察的肿瘤患者于治疗前CD3^+^、CD4^+^、CD4^+^/CD8^+^指标的计数较对照组明显下降，CD8^+^ T淋巴细胞水平明显增高，这与Yan和吴珊珊研究的结果^[8, 11]^相一致。T淋巴细胞亚群指标的变化，提示恶性肿瘤患者的细胞免疫处于免疫抑制状态，机体的正向免疫细胞数量减少，负向免疫细胞增加，体内细胞免疫功能急剧下降，免疫抑制因子过度表达，免疫抑制微环境由此形成，这为肿瘤细胞的免疫逃逸和恶性增殖创造了条件。

本研究结果还显示，在帕博利珠单抗治疗后CD3^+^、CD4^+^、CD4^+^/CD8^+^指标数值较治疗前出现明显升高现象，CD8^+^ T细胞指标数值较治疗前显著降低，证明帕博利珠单抗可能在一定程度上缓解了机体的免疫抑制状态，对机体的免疫系统具有正向调节作用，促进了机体免疫细胞的激活，增强机体的抗肿瘤作用。同时观察还发现治疗第1周期的淋巴细胞亚群较治疗前的变化不具有统计学差异，第2周期仅CD8^+^的变化有统计学意义，而第4周期变化差异具有统计学意义，这可能与免疫治疗起效过程缓慢有关。

与此同时，本研究还比较了帕博利珠单抗治疗后疗效评价为SD/PR患者的淋巴细胞亚群变化与疗效达到PD患者的淋巴细胞亚群变化，发现在治疗后第4周期，疗效达到SD/PR患者的淋巴细胞亚群恢复效果好，但是治疗后疗效达到PD患者的淋巴细胞亚群变化较治疗前没有明显的统计学差异（*P* > 0.05）。结果提示，非小细胞肺癌患者接受帕博利珠单抗治疗后，治疗疗效好的患者的机体免疫抑制状态得到了显著改善，T淋巴细胞浸润增加，抗肿瘤的免疫抑制细胞数目也出现下降，增强了机体抗肿瘤免疫反应，有利于肿瘤患者预后和生存；治疗效果不佳的患者体内的免疫功能未能得到有效的恢复。同时本文通过比较PD-L1不同表达及不同病理学类型患者的疗效显示，PD-L1表达及病理学类型对免疫治疗效果的影响不明显。通过进行单因素与多因素相结合的分析，定期检测免疫治疗患者的T淋巴细胞亚群，对预测疗效具有一定的价值。同时，这为研究在恶性肿瘤发生过程中，淋巴细胞的变化及细胞免疫的状态的改变，提供了一定程度的参考价值。在观察组所有患者中未见明显的不良反应。

综上所述，帕博利珠单抗可以调节非小细胞肺癌患者体内的淋巴细胞亚群变化，改善机体的免疫状态，同时为判断机体的免疫状态及预测免疫治疗效果提供了重要参考价值。因此，定期监测患者体内淋巴细胞亚群的改变，同时在条件允许的情况下结合免疫相关因子的分析，对患者的治疗方案的选择和预后提供参考依据。本试验目前仅随访至患者进行免疫治疗第4周期，而且本观察组中仅第4周期淋巴细胞亚群的变化较治疗前差异有统计学意义（*P* < 0.05），淋巴细胞亚群是否可以作为免疫治疗疗效的预测指标，还有待验证；同时本观察组中样本量相对有限，因此为求进一步验证试验结果，还需要在临床工作过程中不断收集、总结及分析临床数据。
